# Survival in infants with trisomy 18, palliative care and ethical reflections: a single center considerations

**DOI:** 10.1186/s13052-025-02181-7

**Published:** 2026-01-03

**Authors:** Serena Caggiano, Sabrina Persia, Francesco D’Amore, Marina Macchiaiolo, Maria Fornari, Vitangelo Clemente, Maria Giovanna Paglietti, Alessandra Schiavino, Gianfranco Butera, Sergio Filippelli, Luigi Zucaro, Renato Cutrera

**Affiliations:** 1 Pneumology and Cystic Fibrosis Unit, Bambino Gesù Children’s Hospital, IRCCS, Rome, Italy; 2 Academic Department of Pediatrics (DPUO), Bambino Gesù Children’s Hospital, IRCCS, Rome, Italy; 3 Bocconi University, BIDSA, Milan, Italy; 4 Rare Diseases and Medical Genetics Unit, Bambino Gesù Children Hospital, IRCCS, Rome, Italy; 5 Bioethical Function, Bambino Gesù Children’s Hospital, IRCCS, Rome, Italy; 6 Emergency Unit, Acceptance and General Pediatrics, Bambino Gesù Children’s Hospital, IRCCS, Rome, Italy; 7 Paediatric Cardiac Surgery Unit, Bambino Gesù Children’s Hospital, IRCCS, Rome, Italy

**Keywords:** Trisomy 18, Palliative care, Best interest of patient, Burden of disease

## Abstract

**Background:**

Trisomy 18 was once considered a fatal diagnosis due to the presence of cardiac and extracardiac lesions. However, with the increasing use of therapeutic management, 3% to 25% of infants with trisomy 18 may survive beyond their first year, depending on the interventions provided. Currently, there are no clear and widely accepted criteria to guide medical decisions for children with trisomy 18. This means that patients could often be at risk of either over-treatment or therapeutic abandonment. We aimed to explore the effectiveness of intensive and non-intensive treatments in enhancing the clinical burden of disease and survival of children with trisomy 18 syndrome

**Methods:**

a retrospective monocentric study in Bambino Gesù Children’s Hospital, IRCCS Rome, Italy. We enrolled all patients discharged from our hospital with genetic diagnosis of trisomy 18 between 2018 and 2023. Clinical data from birth were collected and categorized into two groups: those who received intensive treatment and those who underwent a palliative approach. Intensive treatment was defined as corrective heart surgery, use of invasive respiratory support, or at least one hospitalization in an intensive care unit. Survival probabilities at different age intervals were calculated, and the clinical burden of disease was assessed, taking into account device dependence, number of emergency department visits per year, and the daily intake of medications at home

**Results:**

32 patients were enrolled. Children with a low device dependence had significantly higher survival(p= 0,01). Neither palliative nor corrective heart surgery affected survival for patients with major cardiac defects. Conversely in children with minor heart defects surgery significantly increased survival probability(p= 0.01), particularly the corrective approach(p= 0.01). High number of emergency department visits(p=0.03) and high number of drugs taken daily(p=0.02) significantly reduced survival. No significant differences emerged between the two groups in terms of burden of disease.

**Conclusions:**

proportional to the initial clinical conditions all treatment options, which may include both comfort care and heart surgery, should be re-evaluated to determine the approach that prioritizes the best interest of each child with trisomy 18.

## Introduction

Trisomy 18 is one of the most common aneuploidies, after trisomy 21 [1], associated with congenital anomalies resulting in significant complications in the prenatal and postnatal life stages. Trisomy 18 has an estimated prevalence of 1 in 6000 live births [2, 3]. After prenatal diagnosis, 60–80% undergo elective pregnancy termination [4]. Infants born with trisomy 18 usually have several physical abnormalities. Almost 85% of these babies have congenital heart defects, including conditions like atrial and ventricular septal defects, tetralogy of Fallot, and patent ductus arteriosus. [5]. They may also present respiratory, neurological, neoplastic, genitourinary, abdominal, otolaryngologic, and orthopedic complications resulting in a low survival rate in the first months of life. Common causes of death include central apnea or end-organ dysfunction, such as pulmonary hypertension or heart failure [6]. Previously, doctors have considered trisomy 18 to be a fatal diagnosis due to the presence of cardiac and extracardiac lesions. In fact, the American Academy of Pediatrics, twenty years ago, recommended considering withholding neonatal resuscitation in confirmed trisomy 18 cases. [7]. Thanks to the increasing use of therapeutic management for these newborns, a larger number of them are now surviving past their first year of life [8]. Recent estimates suggest that between 3 and 25% of infants with trisomy 18 may survive beyond their first year, depending on the extent of screening procedures and interventions provided [1, 9].

Previously, it was common to only provide comfort care and avoid technological interventions for infants with trisomy 18. However, current literature shows a shift towards offering and choosing technological interventions, such as cardiac surgery, gastrostomy tubes and tracheostomy for these infants with a consequent significant decrease in patient mortality rates [10–12]. When dealing with this new consideration, it is vital to prioritize the well-being of the child to ensure a high standard of living for both the child and their family. Additionally, it’ crucial to respect family’s values [13], sharing the health care decision process with health care providers. Clinicians should accompany children affected by trisomy 18, offering appropriate assistance at all stages of their life in a way that respects and promotes the intrinsic human dignity and the highest worth of their existence [14]. This means sometimes taking action and sometimes refraining from doing so.

Our objective was to explore the effectiveness of intensive and non-intensive treatments in enhancing the burden of disease and survival of children with trisomy 18 syndrome. Based on our findings, we aim to recommend the most appropriate management approach that prioritizes the best interest of the children.

## Methods

We conducted a retrospective monocentric study at Bambino Gesù Children’s Hospital in Rome, Italy, enrolling all patients diagnosed with trisomy 18 from 2018 to 2023. The study excluded cases of partial trisomy 18 due to their distinct clinical evolution in terms of long-term survival and comorbidities. Cases of pregnancy termination resulting from prenatal diagnosis of trisomy 18 were not included in the analysis. Data collected encompassed prenatal diagnosis, genetic features, birth weight, gestational age, gender, age at the time of data collection for living patients, age of death if occurred, and days of hospitalization in the neonatal intensive care unit (NICU). Normal birth weight was defined as 2500 – 4000 grams (gr), low birth weight as 1500 – 2500 grams, and very low birth weight as less than 1500 grams. Preterm gestational age was considered to be less than 37 weeks. The study also recorded congenital heart disease, cerebral anomalies, digestive and/or otorhinolaryngologic (ORL) malformations, nephrologic impairment, orthopedic, endocrinological and/or oncological comorbidities. Furthermore, the data included information on heart surgery treatment, ventilation support, invasive mechanical ventilation (IMV), non-invasive ventilation (NIV), orotracheal intubation, tracheostomy, nutrition and device dependence such as nasogastric tube (NGT) and percutaneous endoscopic gastrostomy (PEG), the number of daily medications taken at home, the number of admissions per year, and the length of stay.

Patients were classified into two groups based on the type of care they received: those who underwent intensive treatment, including intensive care (IC), and those who received a palliative approach without intensive care (non-intensive care, NIC). The intensive approach was defined by specific criteria, such as corrective heart surgery, invasive respiratory support, or at least one hospitalization in the intensive care unit (ICU). On the other hand, cardiac surgery was defined palliative when aimed to modify pulmonary blood flow, enhance intracardiac mixing, or rehabilitate a ventricle before definitive surgery, often to improve the quality of life of patients in clinically incurable situations [15]. In contrast, corrective surgery for congenital heart defects aimed to address or treat heart defects present at birth, such as truncus arteriosus, transposition of the great arteries, Tetralogy of Fallot, atrioventricular defects, aortic stenosis, hypoplastic left heart, and aortic coarctation. Less severe defects included minor and atrial/septal or ventricular defects and patent ductus arteriosus. To evaluate the clinical burden of disease in surviving patients, three criteria were identified: dependence on medical devices, the frequency of emergency department visits per year, and the number of medications taken daily at home. We defined low device dependence for patients with no devices or only with PEG, while high device dependence encompassed children requiring PEG and/or ventilation support, including IMV or NIV. A high number of emergency department visits was considered if the patient had three or more visits per year. Finally, a high number of daily medications was defined as three or more medications per day.

## Ethics statement

The study was approved by the ethical committee (code 1684_OPBG_2018). Data were retrospectively analyzed in line with personal data protection policies, informed consent was obtained from parents.

## Statistical analysis

The statistical analysis was performed using the Julia programming language [16]. Statistical differences among variables related to IC/NIC groups were evaluated by the Mann-Whitney test or the Fisher exact test; in the latter case, odds ratios with a 95% confidence interval were also shown. Statistical tests were defined significant when *p* ≤ 0. 05. Furthermore, the unconditional and the conditional survival curves were computed according to the Kaplan-Meier estimate (with a 95% confidence interval) and their significance was established through a log-rank test.

## Results

Between 2018 to 2023, 36 infants with genetic diagnosis of trisomy 18 were discharged. 4 patients with mosaicism were excluded. Data of 32 patients, 25 of whom female were included. According with our definition of IC and NIC, we identified 9 patients who received NIC and 23 patients IC. Among the two groups there was no statistical differences in gender, gestational age, prenatal diagnosis and mean birth weight (Table 1).

**Table 1:** Demographics and birth history of patients with complete trisomy 18

*n* = 32,9,23	All patients	NIC	IC	OR (95% CI)	p
Female (n)	25	7	18	1.03 (0.08–8.4)	0.9
Male (n)	7	2	5	0,97 (0,1–12,5)	0.9
Median GA [min-max]	37 [26 – 40]	36	37		0.7
Prenatal diagnosis (n)	15	5	10	0.6 (0.1–3.8)	0.8
Median Birth Weight [min-max]	1974 [900 - 2850]	2230	1874		0.06

*GA* Gestational Age, values are expressed in weeks. *NIC* Non intensive Care. *IC* Intensive care . 3 values missing for Median GA. Birth Weight values are expressed in grams. [min-max] refers to minimum and maximum values observed expressed as median

**Fig. 1:**
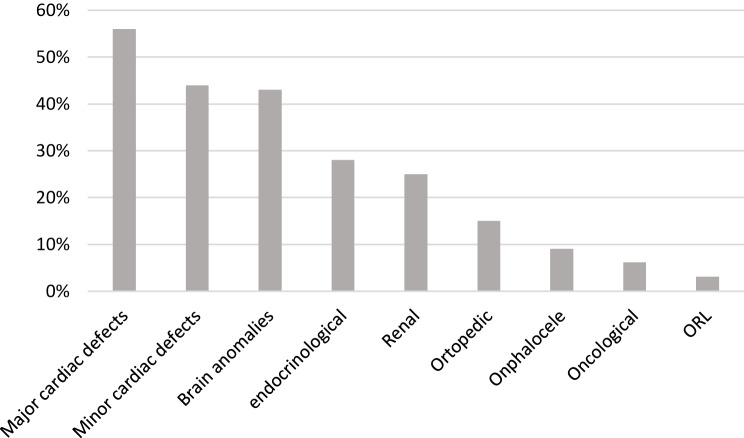
Clinical features of patients with complete trisomy 18. Values are expressed in percentages and refer to the total number of patients. *ORL* otorhinolaryngologic diseases

Out of the total number of patients, 18 had major cardiac defects while 14 had minor heart defects. Brain anomalies were found in 14 patients, while 9 had documented endocrinological involvement. Additionally, 8 children had renal malformations, 5 cases of orthopedic alterations were registered, 3 cases of omphalocele, and 2 patients had oncological complications. Only one child was found to have ORL involvement (Fig. 1).

**Fig. 2:**
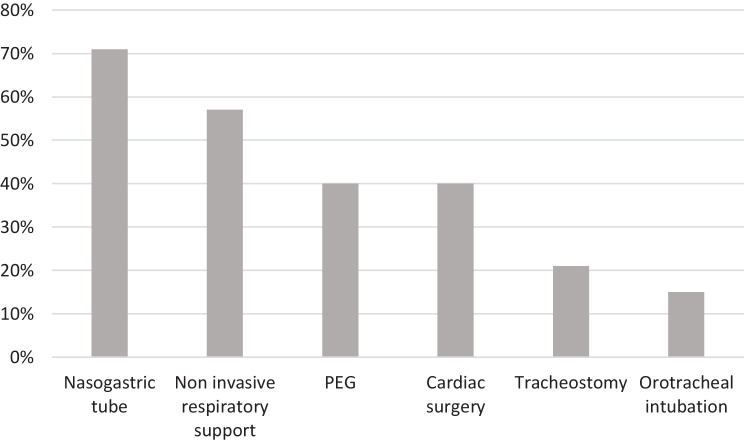
Medical support of patients with complete trisomy 18. Values are expressed in percentages and refer to the total number of patients. *Cardiac surgery* includes both corrective (76%) and palliative surgery (24%)

Figure 2 illustrates the medical support required by our population. Non-invasive respiratory was required by 20 children, while 12 patients needed invasive respiratory support. Among the latter group, 5 underwent orotracheal intubation while 7 underwent tracheostomy.

Among the 32 patients, 24 required enteral feeding. According to our dataset, 13 patients underwent PEG placement. The others were managed with nasogastric tube feeding; 8 patients did not require artificial feeding.

13 out of the 32 patients underwent cardiac surgery, and 10 of them had corrective heart surgery. We analyzed the impact of major or minor heart anomalies on the decision to perform cardiac surgery, but found no statistical significance. Out of the 18 patients with major cardiac defects, 6 received surgical treatment, while out of the 14 patients with minor cardiac defects, 7 were treated surgically (*p* = 0.5).

9 patients died before hospital discharge. Based on the available variables, these patients showed lower gestational age and lower birth weight compared with survivors. Most of them required IC and had major congenital heart defects.

Even if not statistically significant, we registered that the median age at death is higher in children underwent NIC.

We had complete medical reports for only 15/32 children discharged at home after birth and we analyzed their hospital progress, comparing those who received IC to those who received NIC, as shown in Table 2. We evaluated the burden of disease in patients discharged after birth, *n* = 17/32, including also 2 patients followed up at our hospital after a previous period in other medical centers, comparing high device dependence, high number of accesses in emergency department per year and high number of drugs taken daily, no significant differences emerged between the two groups (Table 3).

**Table 2:** Hospital course of patients with complete trisomy 18

	All patients	NIC	IC	*p*
Mean days in NICU (*n* = 25, 5, 20)	62.4	23	72.2	0.06
Mean days per hospitalization (*n* = 15, 3, 12)	12.9	11.5	13.2	0.9
% days/life hospitalization (*n* = 28, 6, 22)	0.6	0.6	0.6	0.9
Mean number of Hospitalizations/year (*n* = 15, 3, 12)	1.8	0.5	2.1	0.07
Number of ICU hospitalizations during life (*n* = 15, 3, 12)	0.2	0	0.3	0.2
Mean age at death (*n* = 23, 5, 17)	809	1415	631	0.5
Median age at death [min-max]	60 [3 - 6990]	74	22	0.06

*NICU* Neonatal Intensive Care Unit. *ICU* Intensive Care Unit. Mean and median age at death is expressed in days. . [min-max] refers to minimum and maximum values observed expressed as median

**Table 3:** Devices dependences. er accesses. Number of drugs used in patients discharged at home after birth in Trisomy 18

*n* = 17, 12, 5	All patients	NIC	IC	OR	*p*
Low devices dependence	12	7	5	0 (0–2.4)	0.3
High devices dependence	5	5	0	inf (0.4-inf)	0.3
Low number of emergency accesses	12	5	7	0 (0–2.4)	0.3
High number of emergency accesses	5	0	5	inf (0.4-inf)	0.3
Low number of drugs	8	4	4	0.1 (0.002–2.07)	0.2
High number of drugs	9	1	8	7 (0.5–442)	0.2

*Low devices dependence* No use of devices or only PEG*. High devices dependence* PEG and mechanic ventilation*. Low number of Emergency accesses* < 3/year*. High number of Emergency accesses* ≥ 3/year*. Low number of drugs* < 3/day*. High number of drugs* ≥ 3*/day*

**Fig. 3:**
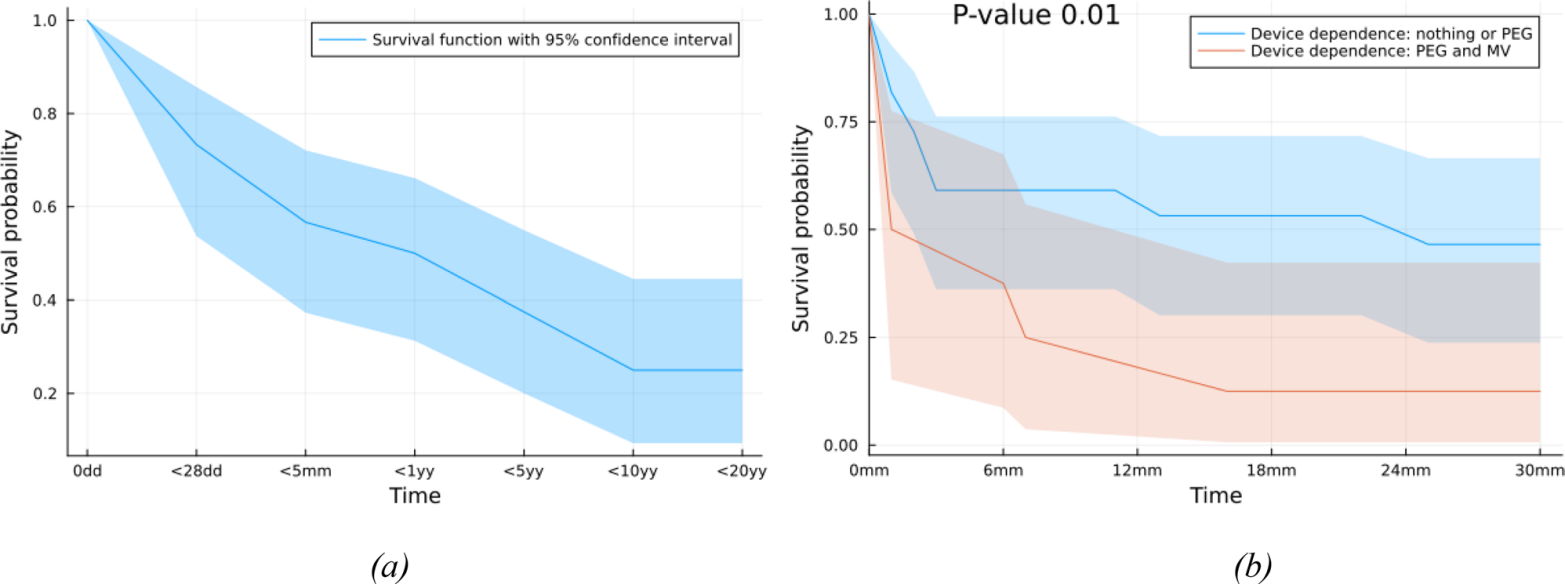
(**a**) Kaplan–Meier 20 years unconditional survival curve (**b**) Kaplan-Meier 30-months survival curve based on devices dependence for infants with complete trisomy 18. *PEG* percutaneous endoscopic gastrostomy. *MV* Mechanic ventilation

In the second part of our study, we estimated unconditional age-specific survival probabilities ( < 1 day, < 7 days, < 28 days, < 5 months, < 1 year, < 5 years, < 10 years, < 20 years) for children with Trisomy 18 (Fig. 3 a).

Survival declined from 73% after the first 28 days to 57% at 5 months, to 50% at 1 year, to 38% at 5 years, to 25% at 10 years. The oldest patient with trisomy 18 lived 19 years and 5 months. 8 patients were living at the completion of the study, the oldest was 6 years old.

Furthermore, we performed Kaplan-Meier survival curves of children with trisomy 18 from 0 months to 30 months and we related them to selected risk factors such as device dependence, birth weight, gestational age, major or minor heart defects and surgery treatment adopted, type of care provided (intensive or non-intensive) and factors we established to evaluate the burden of disease.

Children with low device dependence had significantly higher survival probabilities than those needing more devices than only PEG (*p* = 0,01) (Fig. 3b).

**Fig. 4:**
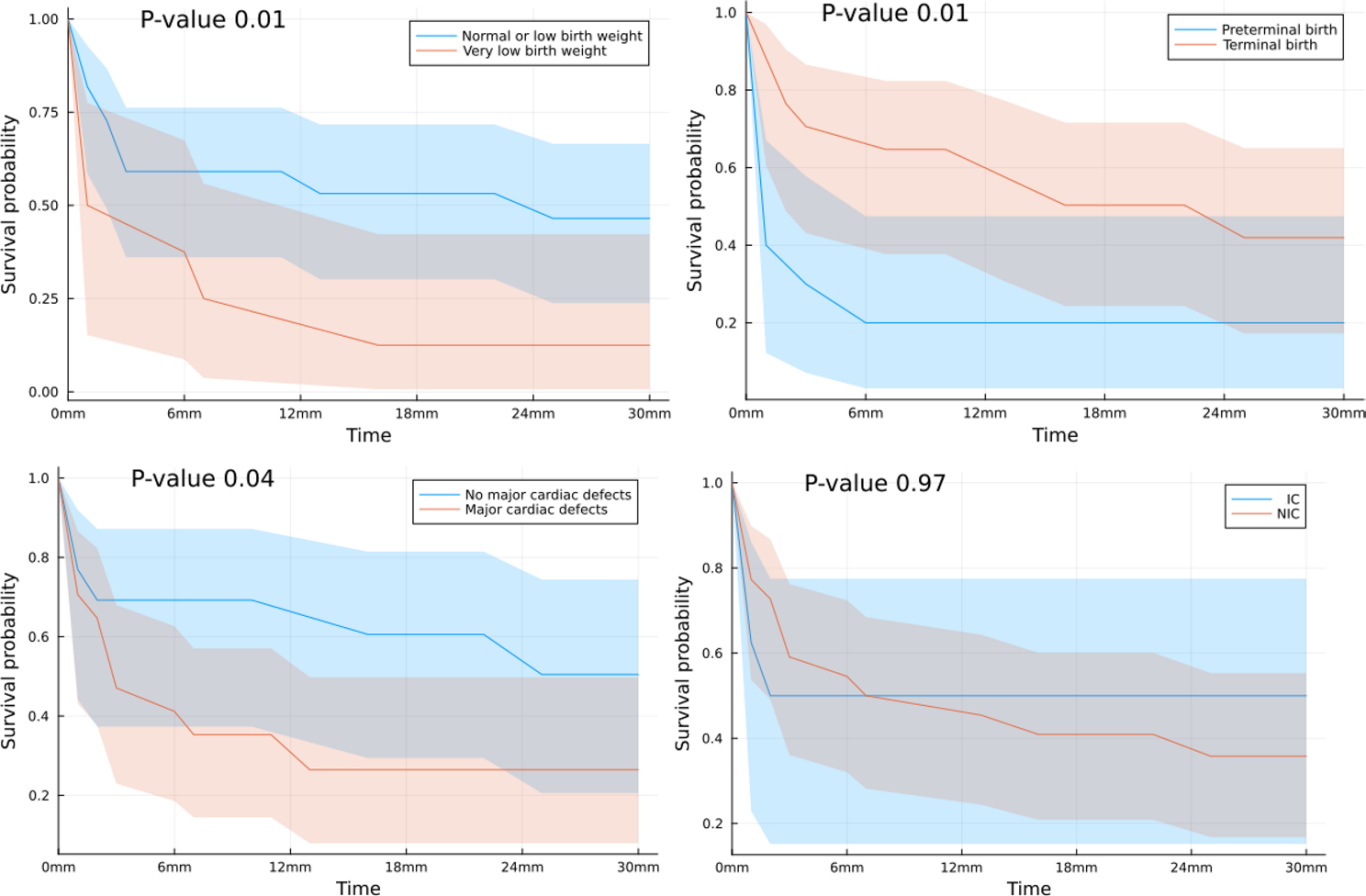
Kaplan–Meier 30 months survival curves for infants with trisomy 18 by selected risk factors. Normal birth weight 2500 g-4000 g . Low birth weight 2500 g-1500 g. Very Low Birth weight <1500 g. Preterminal GA was considered < 37 gestational weeks. mm months. NIC Non intensive care. IC Intensive care.

We found that a very low birth weight (*p* = 0.01) and the preterm birth (*p* = 0.01) significantly affected survival. As expected patients with major cardiac defects had a significant reduction in survival probability (*p* = 0.04), but no statistically significant differences in survival were registered among patients that received IC or NIC (Fig. 4).

**Fig. 5:**
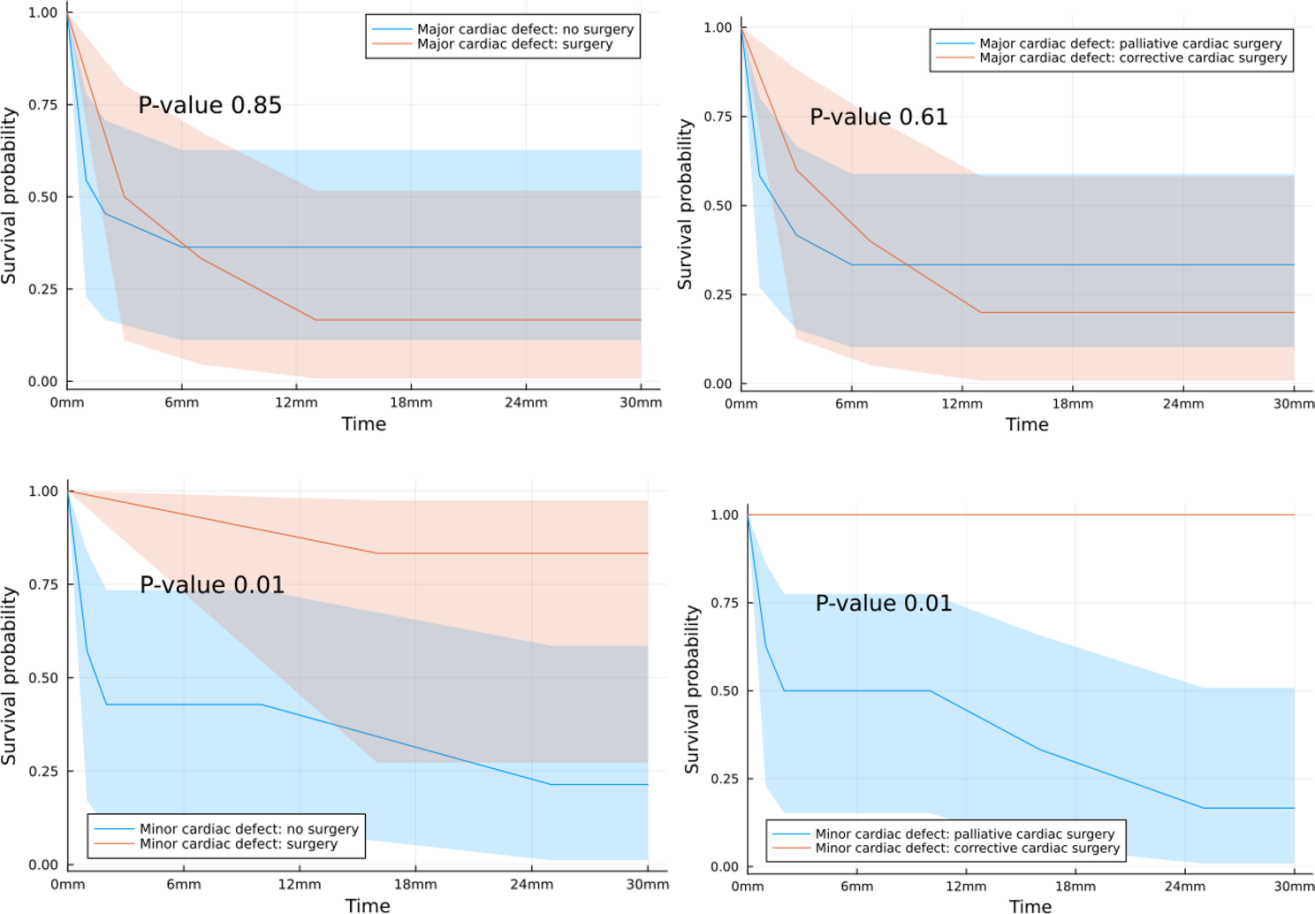
Kaplan–Meier 30 months survival curves for infants with trisomy 18 based on cardiac surgery decision making. *Palliative cardiac surgery* cardiac procedures primarily designed to modify pulmonary blood flow, enhance intracardiac mixing, or rehabilitate a ventricle prior to definitive surgery. *corrective cardiac surgery* definitive fixing heart diseases procedures. *Major cardiac defect* truncus arteries, transposition of great arteries, Fallot tetralogy, atrioventricular defects, aortic stenosis, hypoplastic left heart, aortic coarctation. *minor cardiac defect* atrial or septal cardiac defects

We focused attention on Kaplan–Meier 30 months survival curves for infants with trisomy 18 based on cardiac surgery decision-making (Fig. 5) pointing out that surgery didn’t affect survival for patients with major cardiac defects, neither palliative nor corrective heart surgery. Conversely in children with minor heart defects surgery significantly increases survival probability (*p* = 0.01), particularly the corrective approach more than the palliative cardiac surgery (*p* = 0.01).

Subjects registered with history of high number of accesses in emergency department and high number of drugs taken daily showed significant lower survival probabilities in Kaplan–Meier 30 months survival curves respectively *p* = 0.03 and *p* = 0.02.

For the remaining analyzed factors including prenatal diagnosis, gender and cerebral anomalies, no significant difference in terms of survival was reported (Appendix).

## Discussion

In our retrospective single-center study, we conducted an analysis of the medical history, treatment modalities, factors influencing survival, and overall clinical impact of trisomy 18 in affected individuals. Despite being based on data from a single institution, these findings offer a valuable insight into the current landscape of this rare syndrome.

Our initial objective was to determine if any birth factors influenced the decision to pursue either an intensive or palliative therapeutic approach. The analysis revealed no significant correlations between therapeutic strategies and variables such as gender, gestational age at birth, mean birth weight, and prenatal diagnosis. The prenatal diagnosis did not influence the choice to adopt a more invasive approach. In fact parents of a child with trisomy 18 are often advised against pursuing aggressive interventions due to the perceived severity of the diagnosis [17]. It seems crucial to make decisions about infants care by prioritizing their “best interest” above all else, and combining various principles such as parent preferences based on balanced information and on individual infant physical characteristics and medical condition [18, 19].

Intensive medical treatment such as gastrostomy tube, tracheostomy and cardiac intervention were offered despite the complexity of the clinical condition at a higher rate of infants in our sample.

The strong confessional inspiration at our Institute maybe leads families to expect more life preservation than in other contexts. These circumstances are probably at the origin of a more interventionist attitude towards life-limiting complications and especially heart malformations in these children. However information about socio-economic background, welfare networks, or psychosocial support was not systematically collected. All decisions regarding intensive versus non-intensive care were taken by medical team together with the parents.

Quality of life is difficult to assess in patients with trisomy 18 who survived to leave the hospital because survivors cannot communicate with words [20] so we tried to find objective indicators to measure the burden of disease in children and their families through the level of devices dependence, number of accesses per year in emergency department and drugs number taken daily at home. Most patients who received IC had effectively a high number of accesses per year in emergency department and high number of drugs number taken daily at home denoting stress and greater burden of disease. In our future research, we plan to share with parents measurable and subjective indicators of their child’s quality of life. After receiving the diagnosis, many parents express their primary aim as providing their child with a good life, defined by happiness and love. Often, parents struggle with finding the right balance between doing too little and doing too much. They want to give their child a chance, but not at the cost of burdensome interventions, pain, or a life spent in the hospital [21, 22].

In our population the Kaplan-Meier survival curves of our children with trisomy 18 from 0 months to 30 months didn’t highlight significant difference in survival among patients that receveid IC or NIC but children with low devices dependence showed significantly higher survival probabilities than those needing more devices, such as PEG and NIV or IMV. We reported survival significant decreased for patients with history of high number of accesses in emergency department and high number of drugs taken daily, highlighting the burden of disease of their short life.

Tyler et al. documented provision of congenital heart surgery improves survivorship compared with those who were not offered intervention for congenital heart disease and that nearly 90% of trisomy 18 patients undergoing cardiac surgery survived to discharge [10] but while cardiac surgery may be beneficial in some cases, surgery should not be the primary focus of initial family education and support [23]. We documented patients with major cardiac defects had a significant reduction in survival probability without improvement if treated surgically neither with palliative nor corrective heart surgery.

In contrast children with minor heart defects significantly increased survival probability particularly with the corrective approach more than with the palliative cardiac surgery. These results confirm that cardiac surgery may benefit some children with trisomy 18, as already documented in patients with fewer clinical problems, without respiratory support and older [24]. Our study has shown that corrective heart surgery yields better results in healthier children with minor heart defects. Furthermore, we have found that this surgery improves survival rates and increases the likelihood of children spending more time at home. These findings support the idea that it is not appropriate to completely rule out heart surgery for children with trisomy 18 simply due to the severity of their genetic diagnosis. Rather, each child should be approached on an individual clinical basis and the goals of their family should be taken into consideration to determine the best course of care

## Limitations of the study

Our study has several limitations. First, the number of patients is small because trisomy 18 is a rare genetic disease. Furthermore, not all patients who survived and discharged at birth underwent to the follow-up. In addition, we conducted our research as a single institution, excluding mosaic presentations, thus the sample subjected to our comprehensive analysis is not very extended.

Second, in some cases it was not possible to fully document all the data we intended to describe, because it was not available in the online medical reports.

Our dataset did not include a dedicated variable for cause of death, and retrospective retrieval was not systematically feasible.

Information about socio-economic background, welfare networks, or psychosocial support was not systematically collected.

Finally, we retrospectively attempted to group the medical interventions chosen for the patients into two categories, depending on intensive or non-intensive treatment received but we didn’t know if goals of care had been shared between physicians and parents at the time of diagnosis.

This important point should be considered in future prospective studies, correlating ethical issues to parental hopes and medical choices.

## Conclusions

To conclude, although trisomy 18 was previously viewed as a condition that was not conducive to life and where treatment options were considered unethical and ineffective, new evidence shows that all therapies, including comfort care and heart surgery, can be reevaluated to enhance the quality of life for affected patients. It is crucial to aid parents in determining the best care for their children. Our data evidenced that major congenital heart defects, very low birth weight and preterm birth were markedly associated with lower survival probability. These findings should be highlighted to support counseling of families because clinical variables seem to be the real pillars to personalize the planning of the treatment.

Additional research is imperative to establish a mutual approach between families and healthcare professionals to share parental objectives, values, priorities and to determine the most suitable ethical and clinical approach that benefits the child’s wellbeing.

## Data Availability

The data used for the analysis in this work are available upon reasonable request from the corresponding author.

## Abbreviations

IC: Intensive care
ICU: Intensive care unit
IMV: Invasive mechanical ventilation
NIC: Non-intensive care
NICU: Neonatal intensive care unit
NIV: Non-invasive ventilation
NGT: Nasogastric tube
ORL: Otorhinolaryngologic
PEG: Percutaneous endoscopic gastrostomy
